# A Regulatory Feedback Loop between HIF-1α and PIM2 in HepG2 Cells

**DOI:** 10.1371/journal.pone.0088301

**Published:** 2014-02-05

**Authors:** Zhenhai Yu, Xiaoping Zhao, Yingying Ge, Teng Zhang, Liangqian Huang, Xiang Zhou, Lei Xie, Jianjun Liu, Gang Huang

**Affiliations:** 1 School of Biomedical Engineering, Shanghai Jiao Tong University, Shanghai, China; 2 Department of Nuclear Medicine, Ren ji Hospital, School of Medicine, Shanghai Jiao Tong University, Shanghai, China; 3 State Key Laboratory of Biocontrol, School of Life Sciences, Sun Yat-sen University, Guangzhou, China; 4 Institute of Health Sciences, Shanghai Jiao Tong University School of Medicine (SJTUSM) & Shanghai Institutes for Biological Sciences (SIBS), Chinese Academy of Sciences (CAS), Shanghai, China; Duke University Medical Center, United States of America

## Abstract

To survive under hypoxic conditions, cancer cells remodel glucose metabolism to support tumor progression. HIF transcription factor is essential for cellular response to hypoxia. The underlying mechanism how HIF is constitutively activated in cancer cells remains elusive. In the present study, we characterized a regulatory feedback loop between HIF-1α and PIM2 in HepG2 cells. Serine/threonine kinase proto-oncogene PIM2 level was induced upon hypoxia in a HIF-1α-mediated manner in cancer cells. HIF-1α induced PIM2 expression via binding to the hypoxia-responsive elements (HREs) of the PIM2 promoter. In turn, PIM2 interacted with HIF-1α, especially a transactivation domain of HIF-1α. PIM2 as a co-factor but not an upstream kinase of HIF-1α, enhanced HIF-1α effect in response to hypoxia. The positive feedback loop between PIM2 and HIF-1α was correlated with glucose metabolism as well as cell survival in HepG2 cells. Such a regulatory mode may be important for the adaptive responses of cancer cells in antagonizing hypoxia during cancer progression.

## Introduction

Hypoxia is a hallmark of solid tumor physiology [1]. A hypoxic microenvironment initiates multiple cellular responses, including glycolysis and angiogenesis, which supports cancer development and progression [2]. The homeostatic response to hypoxia is predominantly mediated by a transcription factor hypoxia-inducible factor (HIF). HIF consists of two basic helix-loop-helix proteins of the PAS family, a hypoxia regulated α-subunit (HIF-1α or HIF-2α) and a constitutively expressed β-subunit (HIF-1β) [3]. HIF-1α is one of key regulatory components which can be hydroxylated on proline residues by a class of prolyl hydroxylases under normoxia. Prolyl-hydroxylated HIF-1α is recognized and targeted by an E3 ubiquitin ligase, the von Hippel-Lindau (VHL) tumor suppressor protein [4]. HIF-1α remains unmodified by prolyl hydroxylases under hypoxia, and thereby escapes recognition by VHL and destruction [5]. HIF-1α dimerizes with HIF-1β, and translocates to the nucleus to activate genes in response to hypoxia by binding to the core sequence (RCGTG) of the hypoxic responsive element (HRE) with additional recruitment of the coactivators CBP/p300 [6]. Hydroxylation of HIF-1α at asparagine, which is catalyzed by FIH-1 in normoxic cells, blocks the binding of the transcriptional coactivator p300 to HIF-1α [7]. Proteins encoded by HIF-1α target genes that are involved in key aspects of cancer biology, including cell proliferation and metabolism [8], [9].

PIM2 was first identified in T and B cell lymphomas in mice and is a member of a serine/threonine kinase family of proto-oncogenes, which also includes PIM1 and PIM3 [10]. PIM2 is responsible for cell cycle regulation, cell proliferation and other malignant phenotypes of cancer [11]. PIM2 functions as an oncogene by phosphorylating a wide range of cellular proteins [12]. PIM2 enhances the transcriptional activity of c-Myc and stabilizes the protein by phosphorylating it on Ser-329 [13]. PIM2 phosphorylates the cell cycle regulator p27^kip^ on Thr-157 and Thr-198, promoting its degradation [14]. PIM2 also phosphorylates the pro-apoptotic protein BAD on Ser-112, which reverses BAD-induced cell death [15]. Transgenic mice over-expressing PIM2 are predisposed to T cell lymphomas, whereas PIM2 acts synergistically with c-Myc to accelerate development of B-cell tumors [16]. PIM2 deficiency in mice causes a mild phenotype of reduced body size, impairs proliferation of hematopoietic cells in response to a variety of growth factors, and has an effect on the cell cycle entry of peripheral T cells in response to IL-2 and TCR activation [17]. PIM2 is required for maintaining multiple myeloma cell growth through modulating TSC2 phosphorylation [18]. PIM2 phosphorylates Chk1 and regulates its functions in acute myeloid leukemia [19]. The level of PIM2 is also up-regulated in cancer cells [20].

Here, we characterized a positive feedback loop between PIM2 and HIF-1α. Our data showed that both mRNA and protein levels of PIM2 were remarkably increased in response to hypoxia. And such elevated expression of PIM2 was mediated by HIF-1α, indicating that PIM2 was a direct target gene of HIF-1α. In turn, PIM2 bound to HIF-1α and enhanced its transcriptional activity. Inhibition or over-expression of PIM2 significantly affected hypoxia-stimulated HIF-1α transcriptional activity. Importantly, the up-regulation of PIM2 induced by hypoxia was correlated with cell survival under hypoxia. PIM2 functioned as a co-factor via protein-protein interactions to facilitate the transcriptions of HIF-1α target genes. These results indicate that a crosstalk between PIM2 and HIF-1α through a positive feedback loop regulates hypoxia-induced cellular adaptive response.

## Materials and Methods

### Cell culture and primers

HEK293T, HeLa, A549 and HepG2 cell lines were cultured in DMEM medium supplemented with 10% FBS (Gibco, CA, USA) as well as 100 µ/ml penicillin and 100 µg/ml streptomycin at 37°C and 5% CO_2_. For hypoxia treatment, cells were cultured in a specially designed hypoxia incubator (Thermo Electron, Forma, MA, USA) with 1% O_2_, 5% CO_2_ and 94% N_2_. The primer sequences used in this research are listed in Table S1.

### SiRNA and transfection

Small interfering (si) RNAs against HIF-1α, HIF-2α and PIM2 were purchased from Shanghai GenePharma (china). All transient transfections were performed using Lipofectamine 2000 (Invitrogen) according to the manufacturer's instructions. The siRNA sequences are listed in Table S1.

### Quantitative Real-Time PCR

Total RNA was isolated by TRIzol kit (Omega), and cDNA was synthesized by the cDNA synthesis kit (Takara). Quantitative real-time PCR was performed using the SYBR Green PCR Master Mix (Takara) on the Roche 480 system (Roche).

### ChIP assays

Cells were exposed to hypoxia for 12 h. The cells were analyzed by Chromatin immunoprecipitation (ChIP) assay kits (Upstate, USA) according to the manufacturer's instructions as previously described [21]. The immunoprecipitated DNA fragments were determined by real-time PCR analysis.

### Western blot

Cells were harvested and lysed with ice-cold lysis buffer (RIPA, Sigma). After centrifugation at 12000 g for 10 min at 4°C, proteins in the supernatants were quantified and eluted by the 5×sodium dodecyl sulphate (SDS) sample buffer and boiled 8 min at 100°C, separated by 12% SDS-PAGE and transferred to NC membranes. After blocking with 5% non-fat milk, membranes were immunoblotted with the indicated antibodies. The signals were detected by odyssey imaging instrument according to manufacturer's instructions. The antibodies used are listed in Table S1.

### Co-immunoprecipitation

Cell extracts were incubated with mouse monoclonal or rabbit polyclonal antibodies together with protein A agarose (Pierce) overnight at 4°C. Normal mouse or rabbit IgG (Santa Cruz Biotech) antibodies were used as negative controls. After washing with lysis buffer (RIPA, Sigma) five times, the immunoprecipitates were determined in immunoblot assays using the indicated antibodies [22].

### GST pull-down assay

The GST alone, GST-tagged and His-tagged proteins were purified from *E.coli* BL21 (DE3) system. The GST-tagged proteins were enriched by Glutathione-Sepharose 4B beads (Amersham Biosciences) according to the manufacturer's instructions (Amersham Biosciences, Buckinghamshire, UK). His-tagged proteins were prepared and purified using Ni-affinity resins (Merk). His-PIM2 protein was mixed with GST or GST-HIF-1α (575-826aa) fusion proteins in PBS binding buffer (Takara's PBS, pH 7.4) at 4°C for 2 h, followed by the addition of 20 µl of Glutathione-Sepharose 4B beads. After 1h of mixing, the beads were washed with PBS five times. The pulled proteins were detected by western blots as previously described [22], [23].

### Luciferase Reporter Assays

The PIM2 promoter was amplified from a human genomic DNA template and inserted into the pGL3-promoter vector (Promega). A mutant HIF-1α binding motif was generated using a PCR mutagenesis kit (Toyobo). For luciferase reporter assays, Cells were seeded onto 6-well plates, transfected with the indicated vectors exposed to 1% O_2_ for 24 h. the cell lysates were analyzed by the Dual-Luciferase Assay system (Promega) according to the manufacturer's instructions as previously described [22].

### Glucose Consumption and Lactate Production

Cells were seeded onto 6-well plates and transfected with plasmids or siRNAs. About 48 h after transfection, cells were washed and cultured in a serum-free DMEM medium for a further 16 h. Glucose levels in medium were measured using a glucose assay kit (Sigma), and lactate levels in medium were measured using a Lactate assay kit (CMA, Microdialysis) as previously described [22]. These readouts were normalized to corresponding protein amounts (Thermo Scientific).

### Flow Cytometry

The quantitative analysis of apoptotic cell death was performed using the FITC Annexin V Apoptosis Detection Kit II (Invitrogen). HepG2 cells were transfected with siRNAs. After 24 h, the cells were plated at 1×10^6^ cells/well in a new 6-well plate, incubated under normoxia or hypoxia for a further 24 h, and the cells were analyzed by fluorescence-activated cell sorting (FACS) using an ARIA3 apparatus (BD Biosciences, San Jose, CA, USA).

### Cell Proliferation Analysis

Cells were seeded onto 6-well plates, transfected with scramble siRNA or PIM2 siRNA. After 24 h, 4000 cells were harvest to seed in triplicate in 24-well plates, and cell numbers were counted every 24 h over a period of 3 days [22].

### Statistical analysis

We determined the significance of differences in this research using Pearson's correlation test and Student's t test (two-tailed). *P*<0.05 was considered to be significant. Statistical significance is displayed as *P<0.05, **P<0.01.

## Results

### PIM2 expression is induced by hypoxia

**Figure 1:**
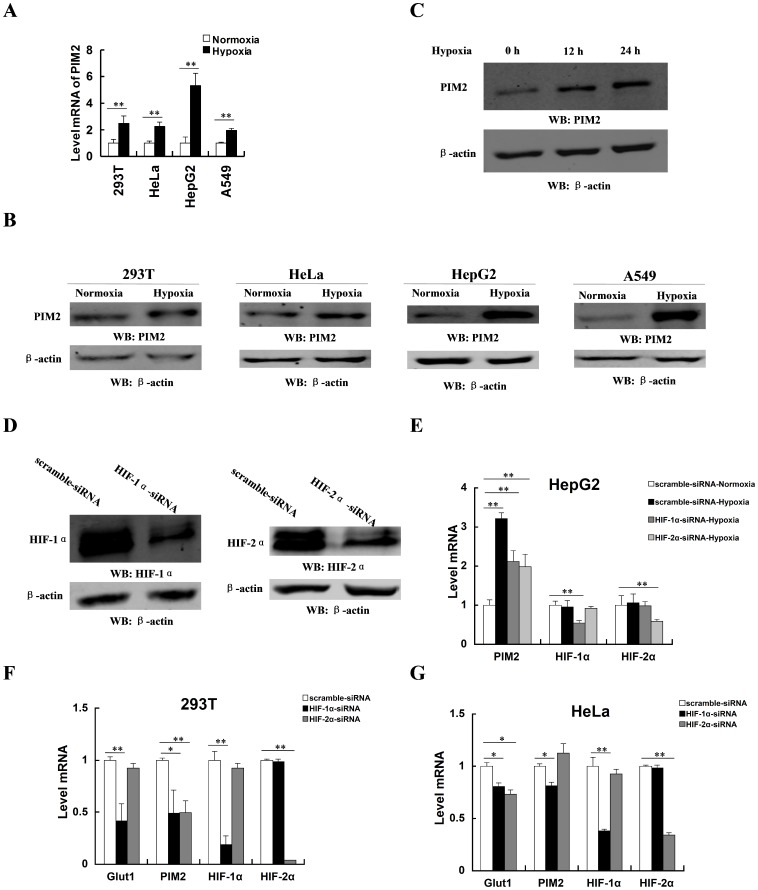
PIM2 expression is induced by hypoxia. (A) mRNA levels of PIM2 in HEK293T, HeLa, HepG2 and A549 cells under normoxia (20% O_2_) or hypoxia (1% O_2_) for 24 h were determined in real-time PCR assays. (B) Protein levels of PIM2 in HEK293T, HeLa, HepG2 and A549 cells under normoxia or hypoxia for 24 h were determined in immunoblot assays using the indicated antibodies. (C) HepG2 cells were cultured under hypoxia for the indicated length of time. Cell lysates were analyzed in immunoblot assays using the indicated antibodies. (D) HEK293T cells were transfected with HIF-1α siRNA or HIF-2α siRNA for 24 h and cultured under normoxia or hypoxia for a further 24 h. Protein levels of HIF-1α or HIF-2α were determined in immunoblot assays using the indicated antibodies. (E) HepG2 cells were transfected with HIF-1α siRNA or HIF-2α siRNA for 24 h and cultured under normoxia or hypoxia for a further 24 h. mRNA levels of PIM2, HIF-1α or HIF-2α were determined in real-time PCR assays. (F and G) HEK293T (F) and HeLa (G) cells were transfected with HIF-1α siRNA or HIF-2α siRNA for 24 h and cultured under hypoxic conditions for a further 24 h. mRNA levels of *PIM2*, *HIF-1α*, *HIF-2α* or *Glut1* (positive control) were determined in real-time PCR assays. All data represent the means ± SEM of three independent experiments, *p<0.05, **p<0.01.

To investigate whether PIM2 expression was oxygen-regulated, HepG2, HeLa, A549, and HEK293T cells were exposed to 20% or 1% O_2_ for 24 h. The mRNA level of PIM2 was increased under hypoxia (Figure 1A). Immunoblot assays indicated that the protein level of PIM2 was also increased while cells were challenged by hypoxia (Figure 1B). Moreover, hypoxia up-regulated the protein level of PIM2 in HepG2 cells in a time-dependent manner (Figure 1C). Since HIF mediates the primary transcriptional response to hypoxic stress, we hypothesized that PIM2 was regulated by HIF. HIF-1α and HIF-2α are broadly expressed in cancer cells, which are accounted for the vast majority of HIF-dependent effects [24]. Knock down of HIF-1α or HIF-2α in HEK293T cells is shown in Figure 1D. Hypoxia-induced PIM2 expression was blunted in the absence of HIF-1α or HIF-2α (Figure 1E). The mRNA level of PIM2 was decreased, while HIF-1α or HIF-2α was knocked down by siRNA in HEK293T cells (Figure 1F). In HeLa cells, the mRNA level of PIM2 was regulated by HIF-1α but not HIF-2α (Figure 1G). Consistently HIF target gene, Glut1, was down-regulated while HIF-1α or HIF-2α was knocked down. Taken together, these results suggest the expression of PIM2 is up-regulated by hypoxia, which is mainly mediated by HIF-1α. In some cases, HIF-2α is also involved in the regulation of PIM2 expression. Since HIF-1α plays a more dominant role in hypoxic response, we focused our research on the study of relationship between HIF1-α and PIM2.

### PIM2 is a HIF-1α target gene

**Figure 2:**
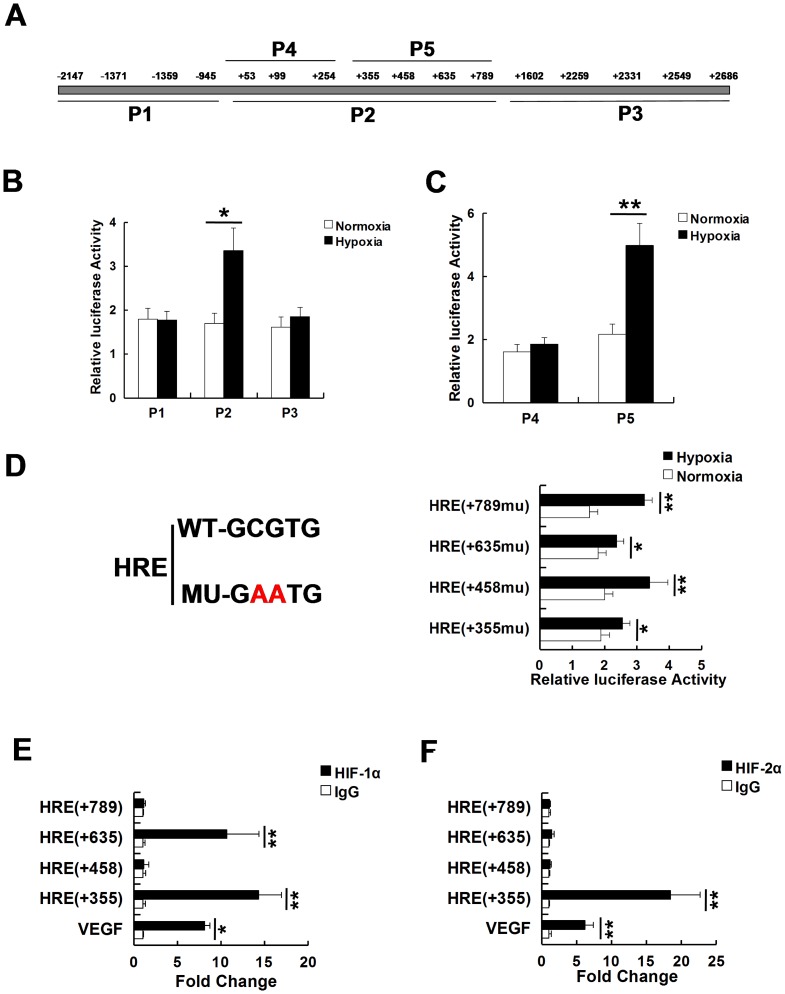
PIM2 is a HIF-1α target gene. (A) Schematic representation of the hypoxic response element (HRE) binding sites in the PIM2 promoter region. Sequences for putative binding sites and their positions were indicated. (B) HEK293T cells were transfected with HRE-luciferase reporter plasmids (P1, P2 or P3) under normoxia or hypoxia. After 48 h, luciferase activity was measured. Transfection efficiency was normalized against Renilla luciferase expression. (C) HEK293T cells were transfected with HRE-luciferase reporter plasmids (P4 or P5) under normoxia or hypoxia. After 48 h, luciferase activity was measured. Transfection efficiency was normalized against Renilla luciferase expression. (D) Schematic diagrams of the regulating sequences with the putative HREs of PIM2. Mut: CG was changed into AA. HEK293T cells were transfected with the HRE (+355mu), HRE (+458mu), HRE (+635mu) or HRE (+789mu)-luciferase reporter plasmids under normoxia or hypoxia. After 48h, luciferase activity was measured. Transfection efficiency was normalized against Renilla luciferase expression. (E and F) Anti-IgG, anti-HIF-1α (E) or anti-HIF-2α (F) antibodies were used in the chromatin immunoprecipitation (ChIP) assays using HEK293T cells which were treated with hypoxia for 12 h. All data represent the means ± SEM of three independent experiments, *p<0.05, **p<0.01.

We next tested whether *PIM2* gene was directly regulated by HIF-1α. Analysis of human *PIM2* gene sequence revealed 16 HREs in its promoter region. The potential HREs were located at −2147, −1371, −1359, −945, +53, +99, +254, +355, +458, +635, +789, +1602, +2259, +2331, +2549 and +2686, relative to the start codon site of PIM2 (Figure 2A). To find out which HRE functioned in response to hypoxia, the fragments (P1, P2, P3) spanning these HREs were subcloned into the luciferase reporter plasmids. The reporter plasmids were transfected into HEK293T cells, which were exposed to 20% or 1% O_2_ for 24 h. The promoter activity of P2, but neither P1 nor P3, was significantly increased in response to hypoxia (Figure 2B). We next cut P2 fragment into two pieces (P4 and P5). The luciferase reporter assays showed only P5 promoter activity was induced by hypoxia (Figure 2C). There were four HREs in the P5 fragment, we therefore performed site-directed mutagenesis to identify which HRE was hypoxia-regulated. The promoter activity of HRE mutant positioned at +355 or +635 was blunted in response to hypoxia, which suggested these two sites were functional HREs (Figure 2D). To determine whether HIF-1α bound to the promoter of PIM2 at these sites, ChIP assays were performed in HEK293T cells. Consistent with mutagenesis assays, HIF-1α enriched at both +355 and +635 HREs (Figure 2E). However, HIF-2α only bound at +355 HRE (Figure 2F). In summary, the data indicate that the *PIM2* gene is directly transcribed by HIF-1α and HIF-2α under hypoxia.

### PIM2 interacts with HIF-1α

**Figure 3:**
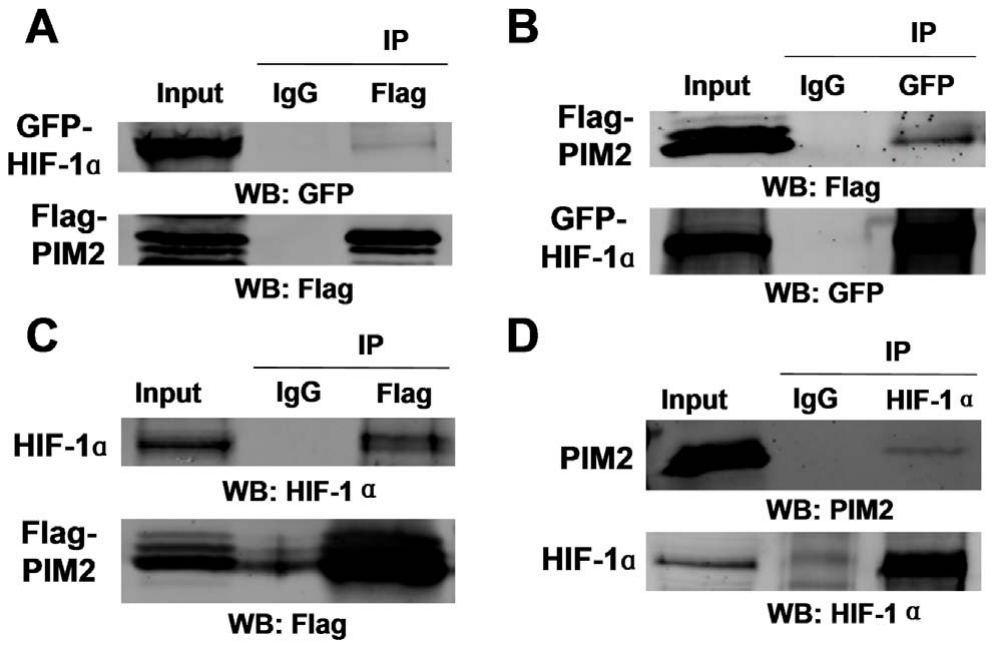
PIM2 interacts with HIF-1α. (A and B) HEK293T cells were transfected Flag-tagged PIM2 with GFP-tagged HIF-1α for 24 h and cultured under hypoxia for a further 24 h. Co-IP assays were performed with anti-Flag (A) or anti-GFP (B) antibodies, followed by immunoblot assays. (C) HEK293T cells were transfected Flag-tagged PIM2 for 24 h and cultured under hypoxia for a further 24 h. Co-IP assays were performed with anti-Flag antibody, followed by immunoblot assays. (D) HepG2 cells were cultured under hypoxia for 24 h. Co-IP assays were performed with anti-HIF-1α antibody, followed by immunoblot assays.

Since PIM2, as a serine/threonine kinase, has been found to regulate transcription factors through mechanisms involving protein-protein interactions and post-translational modulation [25], we hypothesized that a feedback regulation probably existed between PIM2 and HIF-1α. GFP-tagged HIF-1α and Flag-tagged PIM2 were transiently over-expressed in HEK293T cells which were exposed to 1% O_2_ for 24 h. The reciprocal Co-IP data showed that there was an interaction between GFP-tagged HIF-1α and Flag-tagged PIM2 (Figure 3A and 3B). Endogenous HIF-1α was also precipitated by Flag-tagged PIM2 (Figure 3C). Endogenous PIM2 was pulled down by endogenous HIF-1α (Figure 3D). PIM2 also interacted with HIF-2α (Figure S1). PIM2 has been shown to regulate the protein functions by direct phosphorylation [12], so we examined whether PIM2 could phosphorylate HIF-1α. For that purpose, we transfected empty vector or Flag-tagged PIM2 (wild type or kinase dead) with GFP-tagged HIF-1α into HEK293T cells. Compared to empty vector or kinase dead PIM2, transfection of wild type PIM2 had no effect on the serine/threonine phosphorylation levels of HIF-1α as detected by immunoblotting with phospho-Thr/Ser specific antibodies (Figure S3A and S3B), but transfection of wild type PIM2 increased the threonine phosphorylation level of PKM2 (Figure S3C) [22]. To determine whether PIM2/HIF-1α association affected the protein stability of HIF-1α, we manipulated PIM2 expression in HeLa and HepG2 cells. As shown in Figure S2A and Figure S2B, the protein stability of HIF-1α was not significantly affected by PIM2. Taken together, these data demonstrate that the roles of PIM2 on HIF-1α are depended on direct interaction but not phosphorylation.

### PIM2 augments HIF-1α–mediated transcription under hypoxia

**Figure 4:**
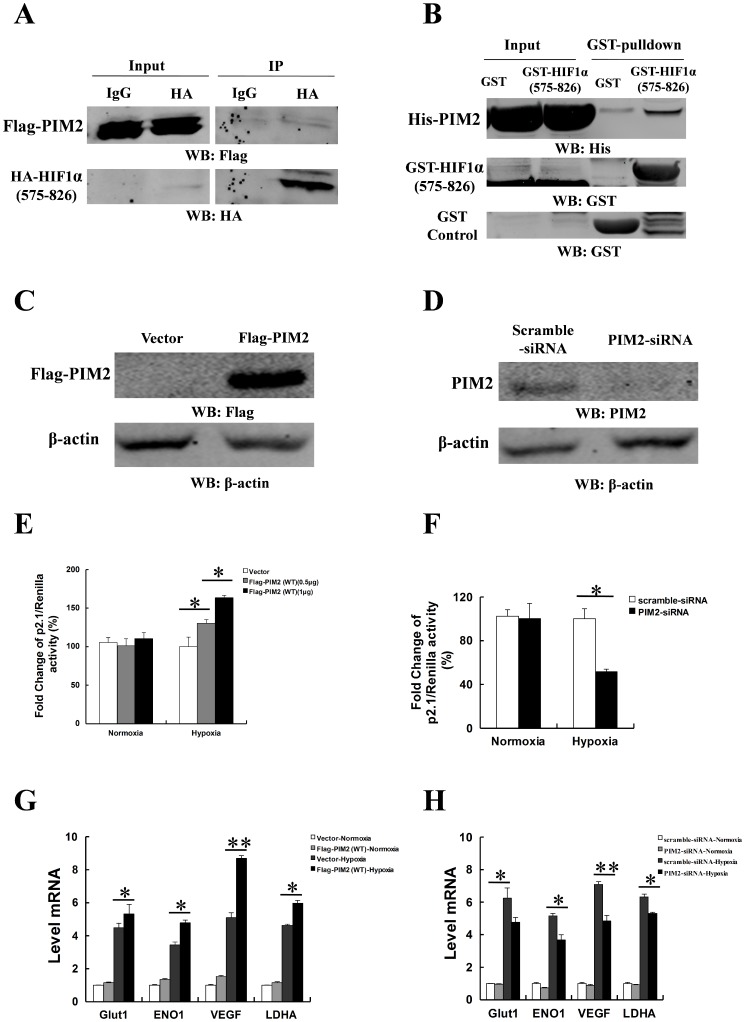
PIM2 augments HIF-1α–mediated transcription under hypoxia. (A) HEK293T cells were transfected Flag-tagged PIM2 with HA-tagged HIF-1α (575-826aa) for 24 h and cultured under hypoxia for a further 24 h. Co-IP assays were performed with anti-HA antibody, followed by immunoblot assays. (B) GST pull-down assays were performed with GST, GST-tagged HIF-1α (575-826aa) and His-tagged PIM2 proteins which were purified from *Ecoli* expressing system. (C and D) HepG2 cells were transfected empty vector or Flag-tagged PIM2 (C); scramble siRNA or PIM2 siRNA (D) for 24 h and cultured under normoxia or hypoxia for a further 24 h. Protein Levels of PIM2 were determined in immunoblot assays using the indicated antibodies. (E and F) HepG2 cells were transfected empty vector or Flag-tagged PIM2 (E); scramble siRNA or PIM2 siRNA (F) with p2.1 luciferase reporter plasmid for 24 h and cultured under normoxia or hypoxia for a further 24 h before luciferase activity was measured. Transfection efficiency was normalized against Renilla luciferase expression. (G and H) HepG2 cells were transfected with empty vector or Flag-tagged PIM2 (G); scramble siRNA or PIM2 siRNA (H) for 24 h and cultured under normoxia or hypoxia for a further 24 h. mRNA levels of *Glut1*, *ENO1*, *VEGF* and *LDHA* were determined by real-time PCR assays. All data represent the means ± SEM of three independent experiments, *p<0.05, **p<0.01.

Since PIM2 interacted with HIF-1α, we analyzed whether their association could regulate transcriptional activity of HIF-1α. HA-tagged HIF-1α transactivation domain (575–826aa) was precipitated by Flag-tagged PIM2 (Figure 4A). It was also confirmed that PIM2 directly interacted with HIF-1a transactivation domain in vitro binding assays (Figure 4B). To test whether PIM2 regulated HIF-1α transcriptional activity, HepG2 cells were co-transfected as following: HIF-1α–dependent reporter plasmid p2.1, which contains HREs from the human *ENO1* gene upstream of SV40 promoter and firefly luciferase coding sequences [26]; control reporter pSV40-Renilla; and scramble siRNA or PIM2 siRNA, empty vector or Flag-tagged PIM2 (wild type or kinase dead). The ratio of p2.1/pSV40-Renilla activity was a specific measure of HIF-1α transcriptional activity [27]. As shown in Figure 4C and 4D, we examined the expression of PIM2 in HepG2 cells while PIM2 was over-expressed or knocked down. Over-expression of Flag-tagged PIM2 (wild type or kinase dead) dramatically increased HIF-1α transcriptional activity in HepG2 cells (Figure 4E and Figure S5A). When PIM2 was down-regulated by siRNA in HepG2 cells, the transcriptional activity of HIF-1α was reduced significantly (Figure 4F). Moreover another two pairs of siRNA targeting PIM2 showed the similar PIM-2 silencing phenotypes in related to the transcriptional activity of HIF-1α (Figure S4). To determine whether PIM2 could regulate the expressions of HIF-1α target genes, HepG2 cells were transfected with empty vector or Flag-tagged PIM2 (wild type or kinase dead), and the transfected cells were exposed to 1% O_2_ for 24 h. Over-expression of PIM2 (wild type or kinase dead) increased expression of the HIF-1α target genes including *Glut1*, *ENO1*, *VEGF* and *LDHA* (Figure 4G and Figure S5B). As shown in Figure 4H, knock down of PIM2 significantly down-regulated the expression of the HIF-1α target genes including *Glut1*, *ENO1*, *VEGF* and *LDHA*. These data suggest that PIM2 is a novel HIF-1α-interacting co-factor, and augments HIF-1α–mediated transcriptional activity under hypoxia.

### PIM2 augments glycolysis and cell survival under hypoxia

**Figure 5:**
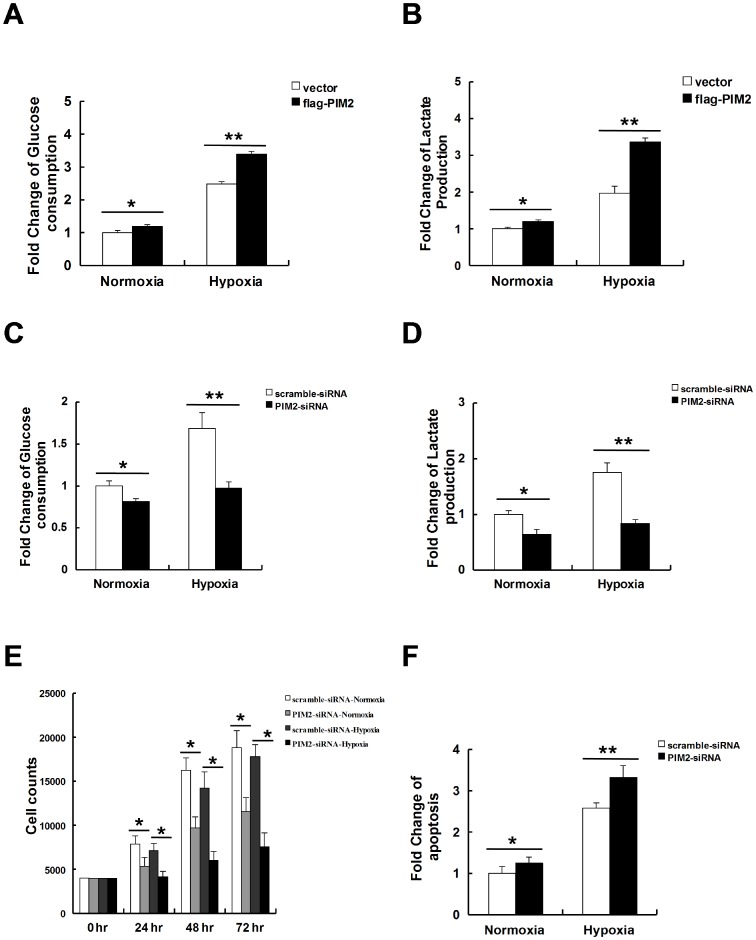
PIM2 augments glycolysis and cell survival under hypoxia. (A and B) HepG2 cells were transfected with empty vector or Flag-tagged PIM2 for 24 h and cultured under normoxia or hypoxia for a further 12–16 h. Levels of glucose (A) or lactate (B) in mediums were determined. (C and D) HepG2 cells were transfected with scramble siRNA or PIM2 siRNA for 24 h and cultured under normoxia or hypoxia for a further 12–16 h. Levels of glucose (C) or lactate (D) in mediums were determined. (E). HepG2 cells were transfected with scramble siRNA or PIM2 siRNA. After 24 h, the cells were re-plated and cultured under normoxia or hypoxia for a further 72 h. Cell numbers were counted every 24 h for the analysis of cell proliferation. (F) HepG2 cells were transfected with scramble siRNA or PIM2 siRNA. After 24 h, the cells were re-plated and cultured under normoxia or hypoxia for a further 24 h. The apoptosis changes were determined using the flow cytometry BD FaCSAria3 apparatus. All data represent the means ± SEM of three independent experiments, *p<0.05, **p<0.01.

Under hypoxia, HIF-1α shunts glucose toward glycolysis to maintain cell survival [28], so we investigated whether PIM2 could augment adaptive responses to hypoxia by increasing glycolysis in cancer cells. Indeed, over-expression of PIM2 increased glucose consumption in HepG2 cells under hypoxia (Figure 5A). Moreover, over-expression of PIM2 also increased lactate production in HepG2 cells under hypoxia (Figure 5B). Consistently, knock down of PIM2 decreased both glucose consumption (Figure 5C) and lactate production (Figure 5D) in response to hypoxia. Compared with cellular responses under normoxia, growth inhibition and apoptosis were increased in the absence of PIM2 while HepG2 cells were challenged with hypoxia (Figure 5E and 5F). Taken together, these data suggest that PIM2 is required for glucose metabolism and cell survival in response to hypoxia.

## Discussion

Tumor hypoxia has been associated with increased malignancy and poor prognosis, promoting extensive research interest into cellular responses to hypoxic challenge for a long time [29]. In this study, we found that both the mRNA and protein levels of PIM2 were significantly induced by hypoxia in human cancer cells. The HIF transcription factors mediate the primary transcriptional responses to hypoxia, so we postulated that HIFs were probably responsible for up-regulating the expression of PIM2 under hypoxia. As expected, hypoxia-induced expression of PIM2 was diminished in the absence of HIF-1α in both HEK293T and HepG2 cells. Similarly HIF-2α was also required for the expression of PIM2 in HEK293T cells but not in HeLa cells. We next used luciferase reporter assays to investigate whether *PIM2* was a target gene of HIF-1α and HIF-2α, the data indicated that HRE sequences positioned at +355 and +635 in the PIM2 promoter region were responsive while cells were challenged with hypoxia. Consistently, ChIP assays validated that HIF-1α bound to HREs positioned at both +355 and +635 in the PIM2 promoter region, whereas HIF-2α only bound to HRE positioned at +355. HIF-1α and HIF-2α have been reported to promote tumor progression through overlapping functions, however, accumulated evidences indicate that HIF-1α and HIF-2α have their unique roles in cancer cells [30]. Here we found that HIF-1α played a major role in regulating PIM2 expression under hypoxia. Even though HIF-1α and HIF-2α are dominantly expressed in cancer cells, our present data could not exclude that the HIF-3α was also involved in PIM2 regulation.

PIM2 has been intensively studied as a protein kinase. Several PIM2 substrates, such as BAD [15], p27 [14] and 4E-BP1 [31], are regulated by post-translational regulations, so we investigated whether HIF-1α was regulated by PIM2 in a feedback manner. Endogenous and exogenous Co-IP assays have indicated an interaction between PIM2 and HIF-1α. GST pull-down assays further confirmed this result. However, no significant difference was found in the protein stability of HIF-1α while PIM2 was either knocked down or over-expressed. Likewise, PIM2 was found to have no effect on the serine/threonine phosphorylation levels of HIF-1α. According to these data, HIF-1α was probably not a substrate of PIM2. Since PIM family members (PIM1, PIM2 and PIM3) are highly conserved [32], they might share the same binding domains. There is a possibility that PIM1 and PIM3 interact with HIF-1α and regulate the protein level of HIF-1α by phosphorylation. And further studies need to decipher their correlation.

Even though the protein level of HIF-1α was not regulated by PIM2, we hypothesized that the functions of HIF-1α were affected after PIM2 binding. Through luciferase reporter assays, we found that the transcriptional activity of HIF-1α was higher in PIM2 over-expression cells. Conversely, the transcriptional activity of HIF-1α was decreased while PIM2 was knocked down. Consistently, the expressions of HIF-1α target genes (*Glut1*, *ENO1*, *VEGF* and *LDHA*) [33] were affected when the expression of PIM2 was manipulated. These data indicate that PIM2, as a HIF-1α target gene, also regulates the transcriptional activity of HIF-1α in a positive feedback manner. Moreover, the roles of PIM2 in this regulatory loop is independent on its kinase activity. This positive feedback signaling may facilitate tumor cells to adapt to hypoxia. More importantly we observed that hypoxia-induced growth inhibition was increased in the absence of PIM2. It suggests that the regulatory feedback loop between PIM2 and HIF-1α may facilitate tumor cells to adapt to hypoxia. Indeed, the feedback mechanisms between HIF-1α and its target genes have been observed in several studies [27], [34]–[36].

The *PIM2* gene is identified as a frequent site for retroviral insertion in experimental Lymphomas [37]. There are multiple isoforms of PIM2 protein (three in the mouse and potentially two in humans) due to the use of the alternative translation start codon, CTG [38]. The multiple isoforms (34 and 41 kDa) have been detected in human cells including primary leukemia cells [39]–[43], myeloma cells [44], [45] and lymphoma cells [46], [47]. 34 kDa PIM2 isoform is mainly analyzed in several studies, which plays an important role in tumor progression [14], [48]. PIM2 mostly functions as an anti-apoptotic/survival factor in normal conditions, but recent studies have indicated that PIM2 has differential functions in different cell lines. PIM2 promotes apoptosis in HeLa cells [48], which is also observed in prostate cancer cells and colorectal cancer cells. The pro-apoptotic role of PIM2 is associated with its distribution in nucleus or cytoplasm [20], [49], [50]. PIM2 has been reported to increase proliferation in HepG2 cells [51], [52], which is consistent with our research. However, we only studied the functions of 34 kDa isoform PIM2 in HepG2 cells, the functions of 41 kDa isoform PIM2 in HepG2 cells need to be further studied in the future.

**Figure 6:**
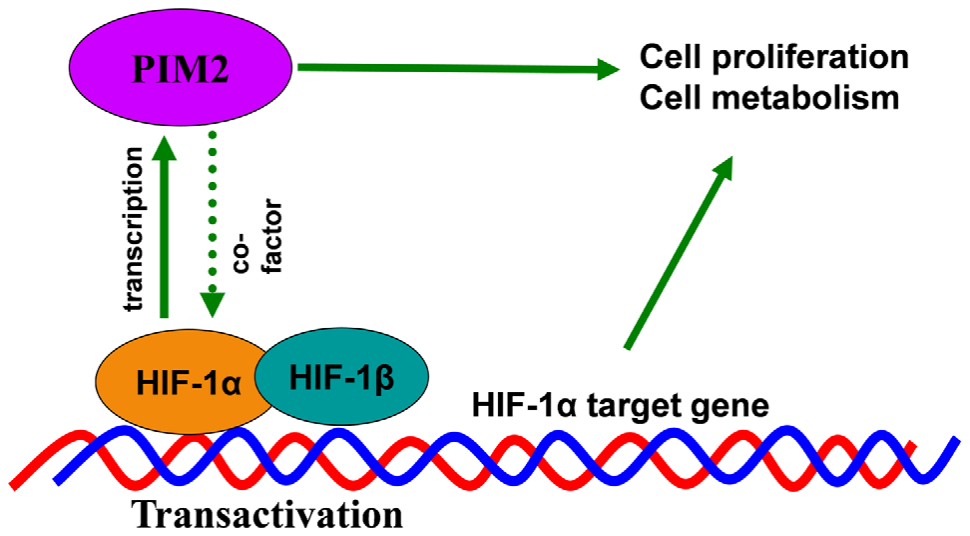
Schematic diagram of the proposed hypoxia-induced PIM2 signaling pathway.

In summary, these discoveries provide new insights into hypoxia-induced expression of PIM2 and PIM2 signaling pathway (Figure 6). Identification of this positive feedback loop may help to understand how intratumoral hypoxia promotes tumor progression. It also provides a theoretical rationale for target PIM2 in cancer therapy.

## Supporting Information

Figure S1 **PIM2 interacts with HIF-2α.** HepG2 cells were cultured under hypoxia for 24 h. Co-IP assays were performed with anti-HIF-2α antibody, followed by immunoblot assays.
(TIF)

Figure S2 **PIM2 has no effect on the protein stability of HIF-1α.** (A and B) HeLa (A) and HepG2 (B) cells were transfected with empty vector or Flag-tagged PIM2; scramble siRNA or PIM2 siRNA. After 24 h, the cells were re-plated and cultured under normoxia or hypoxia. Protein levels were determined in immunoblot assays with the indicated antibodies.
(TIF)

Figure S3 **PIM2 has no effect on the serine/threonine phosphorylation levels of HIF-1α.** (A and B) Effects of PIM2 on the serine (B)/threonine (A) phosphorylation levels of GFP-tagged HIF-1α in HEK293T cells. C. Effects of PIM2 on the threonine phosphorylation level of HA-tagged PKM2 in HEK293T cells.
(TIF)

Figure S4 **Knock down of PIM2 in HEK293T cells and effects of different PIM2 siRNA (s1, s2 or s3) on the transcription activity of HIF-1α in HepG2 cells.** A. HEK293T cells were transfected with scramble siRNA or PIM2 siRNA (s1, s2 or s3). After 48 h, mRNA levels of PIM2 were determined in real-time PCR assays. B. HepG2 cells were transfected scramble siRNA or PIM2 siRNA (s1, s2 or s3) with p2.1 luciferase reporter plasmid for 24 h and cultured under normoxia or hypoxia for a further 24 h before luciferase activity was measured. Transfection efficiency was normalized against Renilla luciferase expression. All data represent the means ± SEM of three independent experiments, *p<0.05, **p<0.01.
(TIF)

Figure S5 **PIM2 (kinase dead) increases the transcription activity of HIF-1α in HepG2 cells.** A. HepG2 cells were transfected empty vector or Flag-tagged PIM2 (kinase dead) with p2.1 luciferase reporter plasmid for 24 h and cultured under normoxia or hypoxia for a further 24 h before luciferase activity was measured. Transfection efficiency was normalized against Renilla luciferase expression. B. HepG2 cells were transfected with empty vector or Flag-tagged PIM2 (kinase dead) for 24 h and cultured under normoxia or hypoxia for a further 24 h. mRNA levels of *Glut1*, *ENO1*, *VEGF* and *LDHA* were determined by real-time PCR assays. All data represent the means ± SEM of three independent experiments, *p<0.05, **p<0.01.
(TIF)

Table S1 Materials used in this research.
(DOC)
