# Supplementary figures and images for: A Regulatory Feedback Loop between HIF-1α and PIM2 in HepG2 Cells
Source: PLoS One. 2014 Feb 5;9(2):e88301. doi: 10.1371/journal.pone.0088301 (PMC3914973; doi:10.1371/journal.pone.0088301)

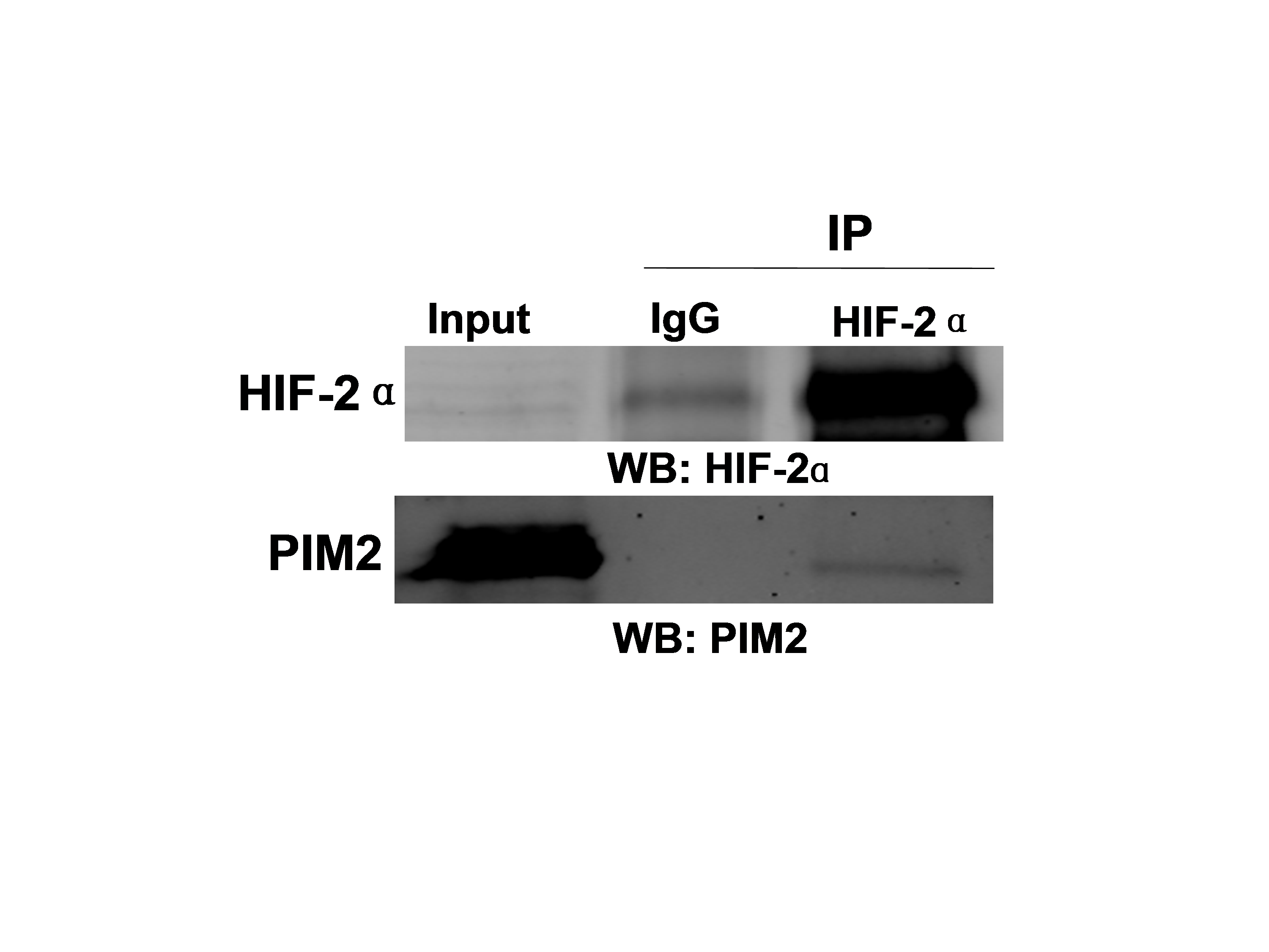

Supplement: Figure S1 — PIM2 interacts with HIF-2α. HepG2 cells were cultured under hypoxia for 24 h. Co-IP assays were performed with anti-HIF-2α antibody, followed by immunoblot assays. (TIF) [file pone.0088301.s001.tif]

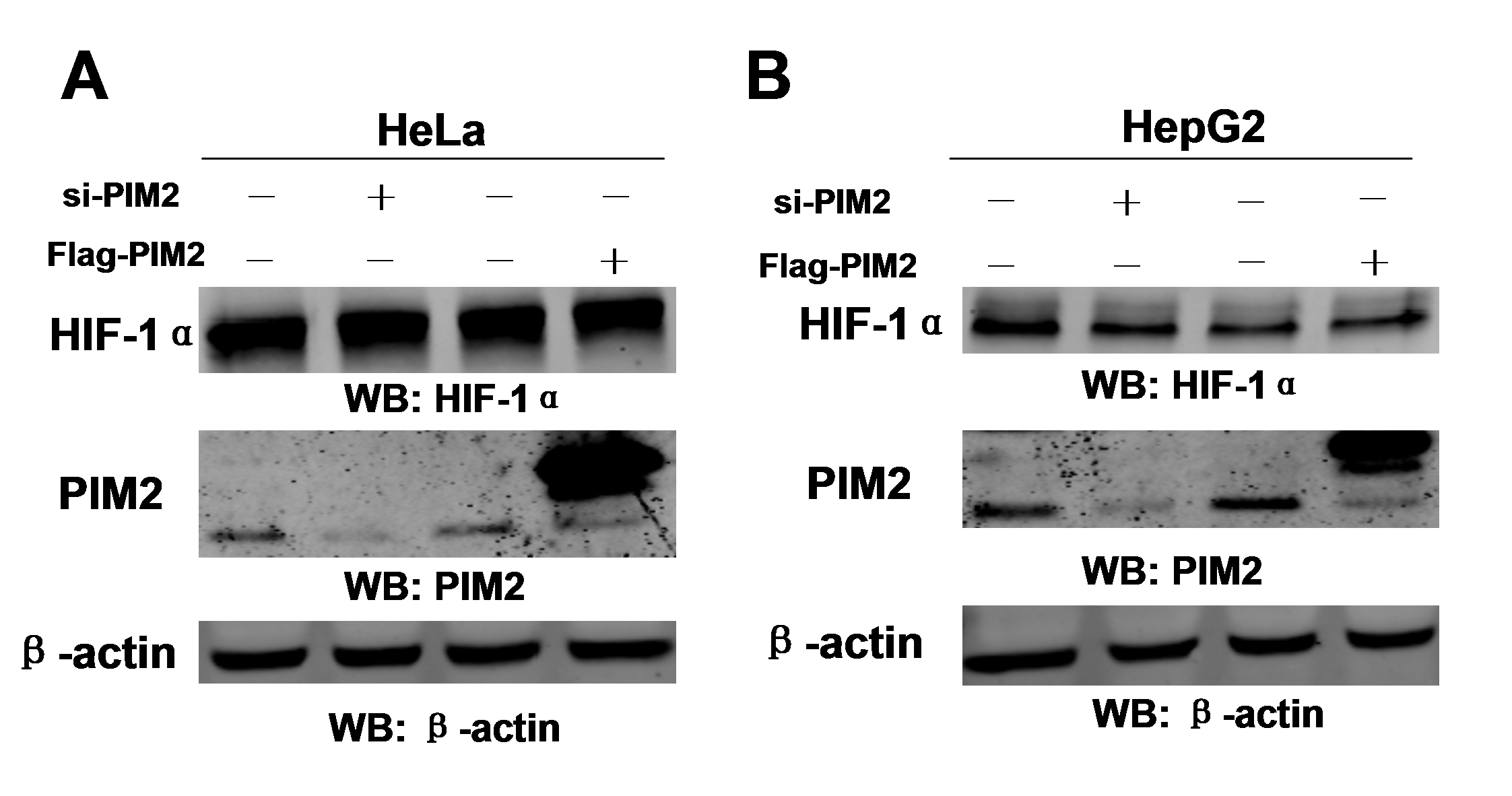

Supplement: Figure S2 — PIM2 has no effect on the protein stability of HIF-1α. (A and B) HeLa (A) and HepG2 (B) cells were transfected with empty vector or Flag-tagged PIM2; scramble siRNA or PIM2 siRNA. After 24 h, the cells were re-plated and cultured under normoxia or hypoxia. Protein levels were determined in immunoblot assays with the indicated antibodies. (TIF) [file pone.0088301.s002.tif]

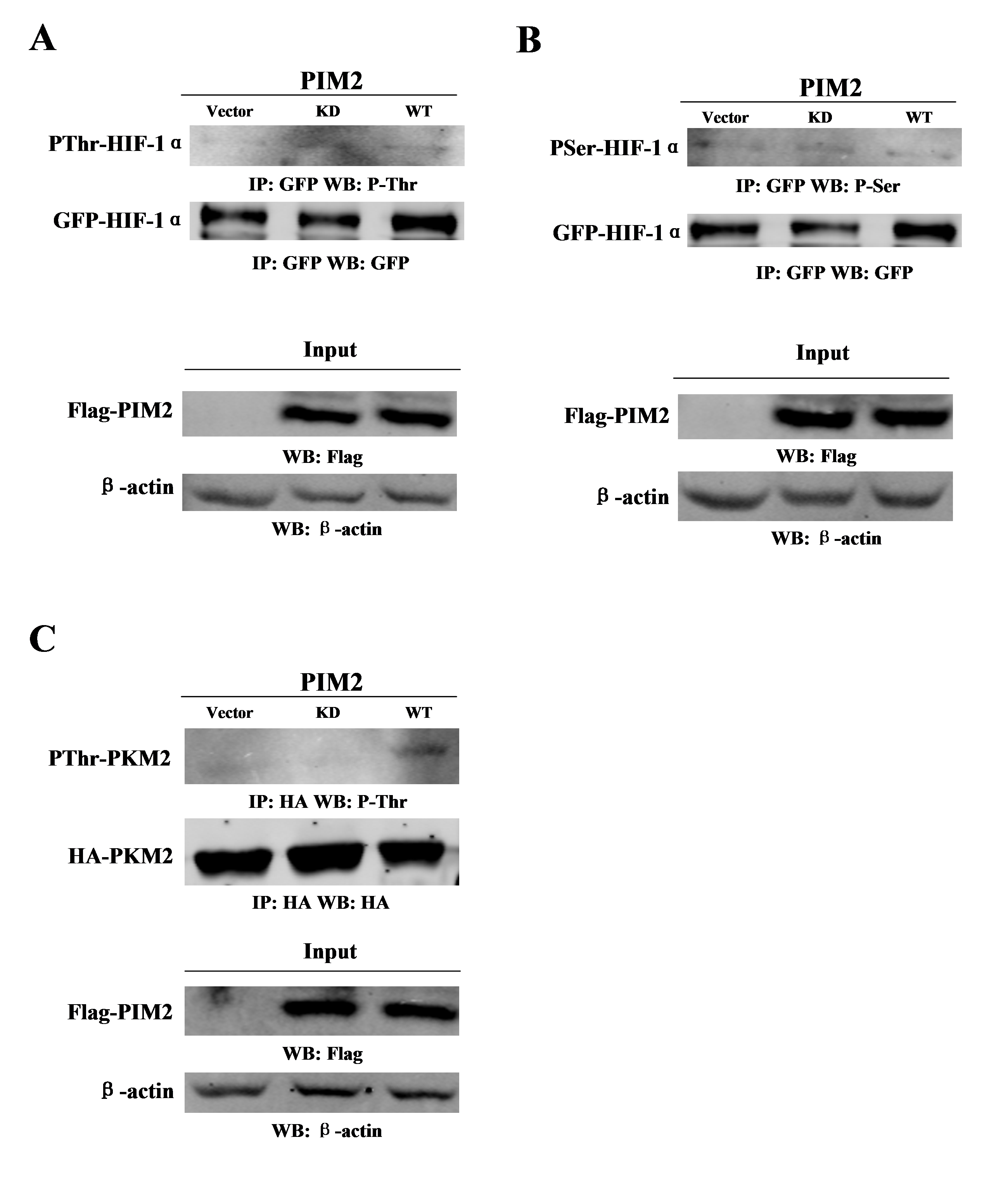

Supplement: Figure S3 — PIM2 has no effect on the serine/threonine phosphorylation levels of HIF-1α. (A and B) Effects of PIM2 on the serine (B)/threonine (A) phosphorylation levels of GFP-tagged HIF-1α in HEK293T cells. C. Effects of PIM2 on the threonine phosphorylation level of HA-tagged PKM2 in HEK293T cells. (TIF) [file pone.0088301.s003.tif]

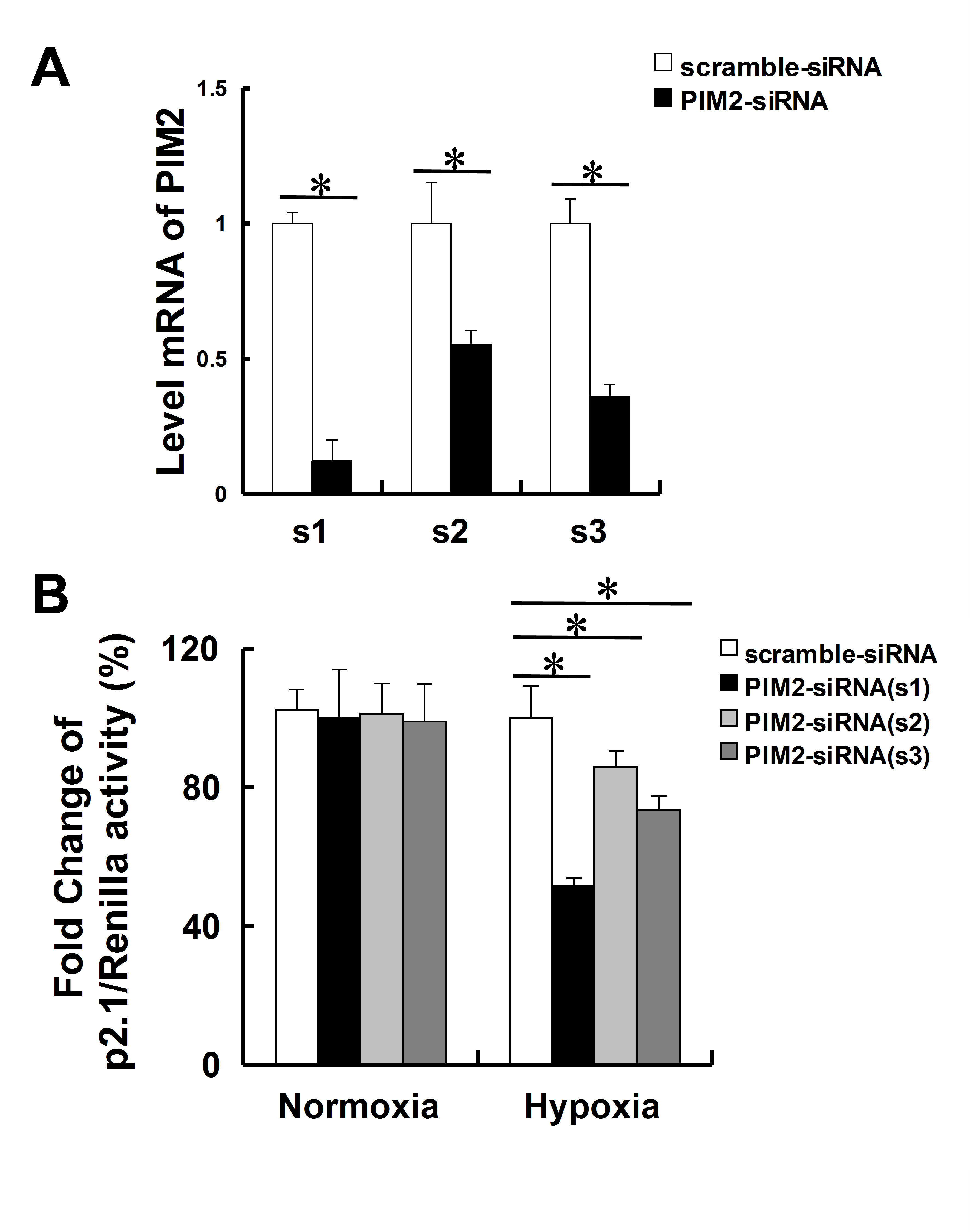

Supplement: Figure S4 — Knock down of PIM2 in HEK293T cells and effects of different PIM2 siRNA (s1, s2 or s3) on the transcription activity of HIF-1α in HepG2 cells. A. HEK293T cells were transfected with scramble siRNA or PIM2 siRNA (s1, s2 or s3). After 48 h, mRNA levels of PIM2 were determined in real-time PCR assays. B. HepG2 cells were transfected scramble siRNA or PIM2 siRNA (s1, s2 or s3) with p2.1 luciferase reporter plasmid for 24 h and cultured under normoxia or hypoxia for a further 24 h before luciferase activity was measured. Transfection efficiency was normalized against Renilla luciferase expression. All data represent the means ± SEM of three independent experiments, *p<0.05, **p<0.01. (TIF) [file pone.0088301.s004.tif]

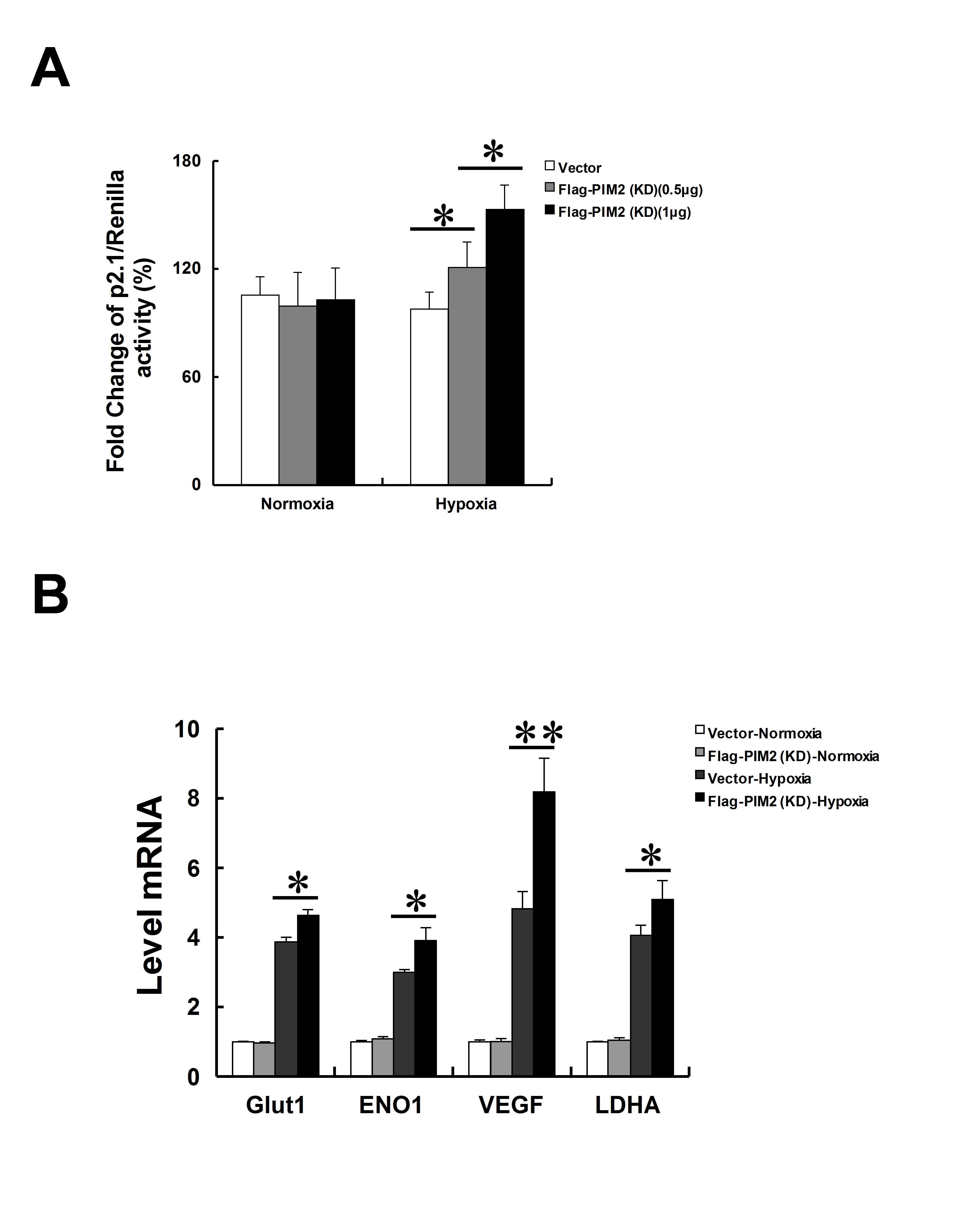

Supplement: Figure S5 — PIM2 (kinase dead) increases the transcription activity of HIF-1α in HepG2 cells. A. HepG2 cells were transfected empty vector or Flag-tagged PIM2 (kinase dead) with p2.1 luciferase reporter plasmid for 24 h and cultured under normoxia or hypoxia for a further 24 h before luciferase activity was measured. Transfection efficiency was normalized against Renilla luciferase expression. B. HepG2 cells were transfected with empty vector or Flag-tagged PIM2 (kinase dead) for 24 h and cultured under normoxia or hypoxia for a further 24 h. mRNA levels of Glut1, ENO1, VEGF and LDHA were determined by real-time PCR assays. All data represent the means ± SEM of three independent experiments, *p<0.05, **p<0.01. (TIF) [file pone.0088301.s005.tif]
