# Supplementary material for: A Regulatory Feedback Loop between HIF-1α and PIM2 in HepG2 Cells
Source: PLoS One. 2014 Feb 5;9(2):e88301. doi: 10.1371/journal.pone.0088301 (PMC3914973; doi:10.1371/journal.pone.0088301)
Supplement: Table S1 — Materials used in this research. (DOC) [file pone.0088301.s006.doc]

**Table S1. Materials used in this research**

| **siRNAs used in this research** | |
| --- | --- |
| scramble-siRNA | 5' UAGCGACUAAACACAUCAA 3’ |
| HIF-1α-siRNA | 5' CUGAUGACCAGCAACUUGA 3’ |
| HIF-2α-siRNA | 5' CAGCAUCUUUGAUAGCAGU 3’ |
| PIM2-siRNA (s1) | 5' CUCGAAGUCGCACUGCUAU 3’ |
| PIM2-siRNA (s2) | 5' GGGGACAUUCCCUUUGAGA 3’ |
| PIM2-siRNA (s3) | 5' CUGCUUCUUUGGCCAAGUA 3’ |
| **Primer sequences used in qRT-PCR assays** | |
| Glut1 | Fw: 5' CAGTTCGGCTATAACACTGGTG 3’  Rev: 5' GCCCCCGACAGAGAAGATG 3’ |
| LDHA | Fw: 5' ATCTTGACCTACGTGGCTTGGA 3’  Rev: 5' CCATACAGGCACACTGGAATCTC 3’ |
| VEGF | Fw: 5' CTTGCCTTGCTGCTCTAC 3’  Rev: 5' TGGCTTGAAGATGTACTCG 3’ |
| ENO1 | Fw: 5’ TGCCGTCTGCAAAGCTGGTG 3’  Rev: 5’ CGCATGGCTTCCCTGAAGTT 3’ |
| HIF-1α | Fw: 5’ CATGTGACCATGAGGAAATG 3’  Rev: 5’ GTTGGTTACTGTTGGTATCATA 3’ |
| HIF-2α | Fw: 5’ ATGACAGCTGACAAGGAGAAG 3’  Rev: 5’ TGTGTTCGCAGGAAGCTGAT 3’ |
| PIM2 | Fw: 5’ ATGTTGACCAAGCCTCTACA 3’  Rev: 5’ TGACTGAGTCTGACAAGGGG 3’ |
| β-actin | Fw: 5’ ATGGATGACGATATCGCTGCGC 3’  Rev: 5’ GCAGCACAGGGTGCTCCTCA 3’ |
| **Primer sequences used in ChIP assays** | |
| chip-PIM2 (+355bp) | Fw: 5' GCAGCGCCACCTCCATGTTG 3’  Rev: 5' ACCCATCATTCCAGCCCACC 3’ |
| chip-PIM2 (+458bp) | Fw: 5' AGACGGGGTGGGCTGGAATGAT 3’  Rev: 5' GGCCTCGAACGCTTCCCGAT 3’ |
| chip-PIM2 (+635bp) | Fw: 5' CAGGACACCGCCTCACAGAT 3’  Rev: 5' CTCACTCCTCGGTCAACCAG 3’ |
| chip-PIM2 (+789bp) | Fw: 5' GTGTGTGTGTCCGTGTCCGT 3’  Rev: 5' ACTCCGGAATCTGAAGCCCC 3’ |
| chip-VEGF | Fw: 5' GCCTCTGTCTGCCCAGCTGC 3’  Rev: 5' GTGGAGCTGAGAACGGGAAGC 3’ |
| **Primer sequences used in expression vectors** | |
| M1 (pGL3-promoter) | Fw: 5' ATGGTACCGTTCATGGTTGTACGTGCCT 3’  Rev: 5' ATCTCGAGCTAGAATGACACCCAGAGAT 3’ |
| M2 (pGL3-promoter) | Fw: 5' ATGGTACCTCCTCGGAAGAACCGAGCGTGTATTTGCAT 3’  Rev: 5' ATCTCGAGCATAACCTCCTAGACCGACA 3’ |
| M3 (pGL3-promoter) | Fw: 5' TAGGTACCACAGTTCTGATTCACCCCAA 3’  Rev: 5' ATCTCGAGTACAAAGGACCTAGCACAAA 3’ |
| M4 (pGL3-promoter) | Fw: 5' ATGGTACCTCCTCGGAAGAACCGAGCGTGTATTTGCAT 3’  Rev: 5' TACTCGAGAGATTGAGCCCACTGAACCC 3’ |
| M5 (pGL3-promoter) | Fw: 5' TAGGTACCATGTTGACCAAGCCTCTACA 3’  Rev: 5' ATCTCGAGCATAACCTCCTAGACCGACA 3’ |
| Mu (+355)-M5 | Fw: 5' CGGGACCCCCATTCCGCCGCCAGGTGAG 3’  Rev: 5' CTCACCTGGCGGCGGAATGGGGGTCCCG 3’ |
| Mu (+458)-M5 | Fw: 5' GGCTGGGCTCAGCATTCCGCCACCCTGACT 3’  Rev: 5' AGTCAGGGTGGCGGAATGCTGAGCCCAGCC 3’ |
| Mu (+635)-M5 | Fw: 5' ACCCCCGCCCCCGCCACATTCACCTGACCCTCCCA 3’  Rev: 5' TGGGAGGGTCAGGTGAATGTGGCGGGGGCGGGGGT 3’ |
| Mu (+789)-M5 | Fw: 5' AGGTGTGTGTGAATGCGAATGTGTGTGTCGGTCTAGGAGGTT 3’  Rev: 5' AACCTCCTAGACCGACACACACATTCGCATTCACACACACCT 3’ |
| P2.1 (pGL3-promoter) | Fw: 5' AAAGGTACCAGGGCCGGACGTGGGGCCCC 3’  Rev: 5' AAACTCGAGGGGGCTCCGTCACGTACTCC 3 |
| PIM2 (pFlag-cmv-4) | Fw: 5' GCGAATTCAATGTTGACCAAGCCTCTACAGGGG 3’  Rev: 5' ATGGATCCTTAGGGTAGCAAGGACCAGG 3’ |
| PIM2 (pFlag-cmv-4)(KD) | Fw: 5'-TGGCCATCGCAGTGATTCCC-3’  Rev: 5'-GGGAATCACTGCGATGGCCA-3’ |
| PIM2 (pet28a) | Fw: 5' GCGAATTCATGTTGACCAAGCCTCTACAGGGG 3’  Rev: 5' ATAAGCTTGGGTAGCAAGGACCAGGCCA 3’ |
| HIF-1α (575-826aa) (pCDNA3.0-HA) | Fw: 5' ATATGGATCCTCCTTCGATCAGTTGTCACC 3’  Rev: 5' CGCGGAATTCTCAGTTAACTTGATCCAAAG 3’ |
| HIF-1α (575-826aa) (pGEX-4T-1) | Fw: 5' ATATGGATCCTCCTTCGATCAGTTGTCACC 3’  Rev: 5' CGCGGAATTCTCAGTTAACTTGATCCAAAG 3’ |
| **Antibodies and beads used in this research** | |
| anti-PIM2 antibody | GneneTex #GTX113928 |
| anti-His antibody | Abmart #2A8 |
| anti-GFP antibody | GneneTex #GTX113617 |
| anti-flag antibody | GneneTex #GTX115043  Abmart #M2008 |
| anti-β-actin antibody | Sigma #A5441 |
| Normal IgG | Santa cruz biotechnology # sc-2025  Santa cruz biotechnology # sc-2027 |
| anti-HIF1α antibody | Novus #NB100-479 |
| anti-HIF2α antibody | Novus #NB100-122 |
| anti-phospho-Serine antibody | Invitrogene # 61-8100 |
| anti-phospho-Threonine Antibody | Cell Signaling # 9381 |
| Goat anti-Mouse  Rabbit second antibody | IRDye 800CW  IRDye 680RD |
| Protein A agarose | Pierce #2033 |
|  |  |
